# Concomitant pulmonary tuberculosis and tuberculous appendicitis in a recipient of a renal transplant: a case report

**DOI:** 10.1186/1752-1947-5-191

**Published:** 2011-05-20

**Authors:** Mohammad R Ardalan, Mohammadali M Shoja, Kamyar Ghabili

**Affiliations:** 1 Department of Nephrology, Tabriz University of Medical Sciences, Tabriz, Iran; 2 Tuberculosis and Lung Disease Research Center, Tabriz University of Medical Sciences, Tabriz, Iran

## Abstract

**Introduction:**

Tuberculosis is still a serious infection among recipients of renal transplants. Although the ileocecal region is the most affected part in intestinal tuberculosis, acute tuberculous appendicitis is quite a rare entity. We report a case of concomitant pulmonary tuberculosis and tuberculous appendicitis in a recipient of a renal transplant.

**Case presentation:**

A 27-year-old Iranian woman, who had been the recipient of a renal transplant five years earlier, presented with a two-week history of coughing, fever and weight loss. The cause of her end-stage renal disease was chronic pyelonephritis. There were fine crackles noted during a chest examination, and a plain chest radiography showed fine miliary nodules throughout her entire lung fields. Sputum and bronchial aspirate examination was positive for acid-fast bacilli, suggestive of *Mycobacterium tuberculosis *infection. A chest computed tomography scan revealed widespread miliary nodules, compatible with miliary tuberculosis. She developed severe abdominal pain and abdominal surgery disclosed a perforated appendicitis. Histopathological examination of the resected appendix revealed widespread caseating epithelioid granulomas, suggestive of tuberculosis.

**Conclusion:**

Our case report highlights a rare presentation of tuberculosis in a patient who has undergone renal transplant. Such unusual presentation of tuberculosis, particularly among patients receiving potent immunosuppressive protocols, should be considered by clinicians.

## Introduction

Tuberculosis (TB) remains a major health hazard particularly in developing countries [1]. Renal transplant recipients are at high risk of a number of infections including mycobacterial diseases [2,3]. The cumulative incidence of post-transplant TB in developing countries is as high as 13.3% with a relatively high rate of extra-pulmonary TB. Although the ileocecal region is the most affected part in gastrointestinal TB, acute tuberculous appendicitis is quite a rare entity [4]. Here, the authors report a renal transplant recipient who developed concomitant pulmonary TB and tuberculosis appendicitis.

## Case report

A 27-year-old Iranian woman, recipient of a living, unrelated renal transplant five years earlier, presented with a productive cough, weight loss and fever. The cause of her end-stage renal disease was chronic pyelonephritis. Six weeks prior to her admission, her immunosuppressant regimen was switched from oral cyclosporine (100 mg, twice daily) to oral sirolimus (1 mg, twice daily) with a diagnosis of chronic allograft nephropathy (serum creatinine level of 2 mg/dL). Her mycophenolate mofetil dose was decreased from 2000 to 1000 mg/day and prednisolone was continued with the previous dose (5 mg, daily). Four weeks later, she developed a mild cough, weakness and anorexia. Her condition progressed to a more constant cough and weight loss until she was hospitalized.

Physical examination revealed her blood pressure to be 110/60 mm/Hg, respiratory rate 20/min, pulse rate 92/min and body temperature 38°C. A chest examination revealed fine crackles mainly on her lower lung fields. On admission, the laboratory findings were as follows: white blood cell count 5.1 × 10^9^/L, hemoglobin 7.2 g/dL, hematocrit 22.4%, mean corpuscular volume 75 fL (normal range, 77-97 fL), mean corpuscular hemoglobin 24 pgm (normal range, 26-32 pgm), mean corpuscular hemoglobin concentration 32% (normal, 32-36%), platelet count 213 × 10^9^/L, fasting blood glucose 105 mg/dL, blood urea 62 mg/dL, serum creatinine 1.9 mg/dL, sodium 135 mEq/L, potassium 4.5 mEq/L, aspartate aminotransferase 37 IU/L, alanine aminotransferase 45 IU/L, alkaline phosphatase 1106 IU/L and total bilirubin 1 mg/dL. A urine analysis was unremarkable.

**Figure 1:**
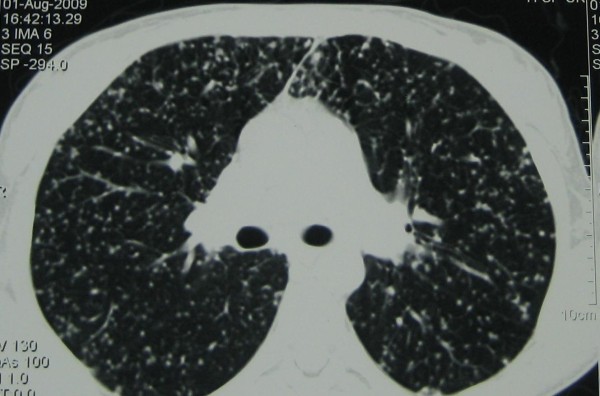
**CT scan shows the typical radiological presentation of miliary tuberculosis**.

On the first day of admission, sirolimus and mycophenolate mofetil were discontinued and cyclosporine (200 mg/day) was started. The dose of prednisolone was not altered (5 mg, daily). A chest X-ray showed fine miliary nodules throughout the entire lung fields. Echocardiography revealed normal pericardium and cardiac chambers. Her left ventricular ejection fraction was 65%. An ultrasound examination of her renal allograft did not reveal any pathologic finding. A 6 mm induration was observed on a TB skin test. Sputum and bronchial aspirate examination was positive for acid-fast bacilli, suggestive of *Mycobacterium tuberculosis *infection. A chest computed tomography (CT) scan illustrated widespread miliary nodules throughout the lung fields, compatible with miliary TB (Figure 1).

**Figure 2:**
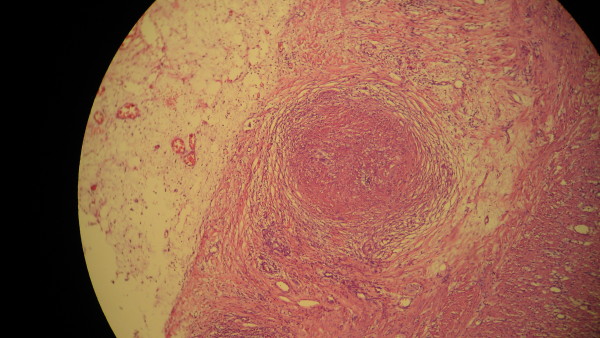
**Histopathologic examination of the resected specimen revealed epithelioid granulomas in sub-mucosa and sub-serosa**. Hematoxylin-eosin staining, 10 × 40 magnification.

In the evening of her fourth day of admission, our patient developed an increasing right lower quadrant abdominal pain spreading to her entire abdomen. Physical examination revealed widespread abdominal tenderness and rebound. An ultrasonographic study of her abdomen disclosed a small fluid collection in the right lower quadrant. Two cysts were detected in her left ovary (54 × 32 mm and 33 × 31 mm). An abdominal CT scan with oral contrast revealed that her pancreas and spleen were normal. Her colonic wall thickness was measured as 4 mm and free fluid was visible in dependent parts of her abdominal cavity. Her peritoneal cavity was then opened with a midline incision and widespread peritonitis was found. Her inflamed appendix (9 × 6 cm) was perforated at its tip. Her ileocecal area and cecum were inflamed with fragile tissues. A cecostomy was performed and a drain was placed. Histopathological examination of the resected appendix revealed caseating epithelioid granulomas, epithelioid histiocytes and Langhans giant cells in the sub-mucosa and sub-serosa of her appendix (Figure 2). The histological picture, along with positive mycobacterial cultures from the sputum and bronchial aspirate, was suggestive of tuberculous appendicitis. After surgery, chemotherapy for TB was started and continued for nine months. An immunosuppressive regimen was continued with cyclosporine, mycophenolate mofetil and prednisolone. During follow-up visits, the pulmonary and abdominal signs and symptoms were resolved.

## Discussion

Our case report showed the occurrence of pulmonary TB and tuberculous appendicitis in a renal transplant recipient. In our patient, TB-induced inflammation, luminal obstruction and superimposing infection were the most likely causes of the acute appendicitis. Tuberculous appendicitis was diagnosed based on the report of caseating epithelioid granulomas in a histopathological examination of the resected appendix, together with positive mycobacterial cultures from the sputum and bronchial aspirate. The definite diagnosis of tuberculous appendicitis requires identification of the causative organism through culture or microbiological evaluation of the resected specimen [5]. Caseating epithelioid granulomas could be reported in a number of fungal infections including histoplasmosis, cryptococcosis, and coccidioidomycosis. Although the two latter fungi can be detected on careful examination of a hematoxylin-eosin stained specimen, histochemical stains such as Grocott's methenamine silver are used most often for identification of these organisms (for review see [6]). Therefore, the fungi infections should be considered in differential diagnoses of TB when caseating necrosis is seen, particularly in immunosuppressive patients.

Renal transplantation increases the risk of TB infection by nearly 50-fold compared to the normal population [7]. Moreover, potential pre-disposing factors for development of post-transplant TB include episodes of rejection [5,8,9], diabetes mellitus [9,10], longer pre-transplant period on hemodialysis [8], chronic liver disease, and co-existing infections [10]. In our patient, however, no predisposing factor was found.

The gastrointestinal system is one of the least prevalent extra-pulmonary sites of TB (3%) which commonly appears in ileocecal region. However, tuberculous appendicitis is a very rare scenario [3]. Although reports indicate an incidence of 0.1 to 3.0% for TB appendicitis in all appendectomies [6,7], prevalence of gastrointestinal TB in renal transplant recipients varies between 0.2% and 0.6% [5]. Extension from the ileocecal region and hematogenous spread are the major proposed mechanisms of appendiceal TB [5]. In non-transplant patients, abdominal pain is the most common presenting symptom of gastrointestinal TB [11]. However, gastrointestinal bleeding followed by fever and abdominal pain was the most prevalent symptom in renal transplant recipients with gastrointestinal TB [5]. Jarrett and colleagues attributed these differences in presentation to decreased inflammatory response in immune-compromised patients [5]. In our case report, the sudden onset of right lower quadrant abdominal pain was suggestive of appendicitis. In addition, since the definite diagnosis of tuberculous appendicitis is mainly based on the granulomatous lesions in post-surgical histopathological examination, pre-operative diagnosis of tuberculous appendicitis may not be clinically feasible [12,13].

## Conclusion

Due to the widespread use of the potent immunosuppressant combinations in the transplant era, clinicians should be familiar with unusual and rare presentations of TB. Concomitant pulmonary TB and tuberculosis appendicitis should be considered as a rare infectious complication in renal transplant recipients.

## Abbreviations

CT: computed tomography; TB: tuberculosis.

## Consent

Written informed consent was obtained from the patient for publication of this case report and any accompanying images. A copy of the written consent is available for review by the Editor-in-Chief of this journal.

## Competing interests

The authors declare that they have no competing interests.

## Authors' contributions

MRA contributed to acquisition of the data and interpreted experiments. MMS and KG interpreted experiments and revised the manuscript. All authors read and approved the final manuscript.

## References

[B1] Ram R Swarnalatha G Prasad N Dakshinamurty KV Tuberculosis in renal transplant recipients Transpl Infect Dis 2007 9 2 97 101 10.1111/j.1399-3062.2006.00182.x 17461993

[B2] Ardalan MR Shoja MM Tubbs RS Ghabili K Transplant renal artery stenosis associated with acute cytomegalovirus infection: resolution following ganciclovir administration Ren Fail 2009 31 10 982 984 10.3109/08860220903288526 20030536

[B3] Ardalan MR Ghaffari A Ghabili K Shoja MM Lepromatous leprosy in a kidney transplant recipient: a case report Exp Clin Transplant 2011 9 3 203 206 21649570

[B4] Singh MK Arunabh Kapoor VK Tuberculosis of the appendix -a report of 17 cases and a suggested aetiopathological classification Postgrad Med J 1987 63 855 857 10.1136/pgmj.63.744.855 3447110 PMC2428607

[B5] Jarrett O Grim SA Benedetti E Clark NM Gastrointestinal tuberculosis in renal transplant recipients: case report and review of the literature Transpl Infect Dis 2011 13 1 52 57 10.1111/j.1399-3062.2010.00540.x 20626712

[B6] Mukhopadhyay S Gal AA Granulomatous lung disease: an approach to the differential diagnosis Arch Pathol Lab Med 2010 134 5 667 690 20441499 10.5858/134.5.667

[B7] Qunibi WY al-Sibai MB Taher S Harder EJ de Vol E al-Furayh O Ginn HE Mycobacterial infection after renal transplantation--report of 14 cases and review of the literature Q J Med 1990 77 282 1039 1060 2267281 10.1093/qjmed/77.1.1039

[B8] Basiri A Hosseini-Moghaddam SM Simforoosh N Einollahi B Hosseini M Foirouzan A Pourrezagholi F Nafar M Zargar MA Pourmand G Tara A Mombeni H Moradi MR Afshar AT Gholamrezaee HR Bohlouli A Nezhadgashti H Akbarzadehpasha A Ahmad E Salehipour M Yazdani M Nasrollahi A Oghbaee N Azad RE Mohammadi Z Razzaghi Z The risk factors and laboratory diagnostics for post renal transplant tuberculosis: a case-control, country-wide study on definitive cases Transpl Infect Dis 2008 10 4 231 235 10.1111/j.1399-3062.2007.00271.x 17655654

[B9] Chen CH Lian JD Cheng CH Wu MJ Lee WC Shu KH Mycobacterium tuberculosis infection following renal transplantation in Taiwan Transpl Infect Dis 2006 8 3 148 156 10.1111/j.1399-3062.2006.00147.x 16913973

[B10] John GT Shankar V Abraham AM Mukundan U Thomas PP Jacob CK Risk factors for post-transplant tuberculosis Kidney Int 2001 60 3 1148 1153 10.1046/j.1523-1755.2001.0600031148.x 11532111

[B11] Horvath KD Whelan RL Intestinal tuberculosis: return of an old disease Am J Gastroenterol 1998 93 5 692 696 10.1111/j.1572-0241.1998.207_a.x 9625110

[B12] Goyal N Khurana N Tuberculosis of the appendix: An unusual occurrence with review of literature ANZ J Surg 2009 79 9 662 19895534 10.1111/j.1445-2197.2009.05032.x

[B13] Siu YP Tong MK Kwok YL Leung KT Kwan TH Lam CS Au TC An unusual case of both upper and lower gastrointestinal bleeding in a kidney transplant recipient Transpl Infect Dis 2008 10 4 276 279 10.1111/j.1399-3062.2007.00286.x 18047566

